# Cancer health awareness through screening and education: A community approach to healthy equity

**DOI:** 10.1002/cam4.7357

**Published:** 2024-06-28

**Authors:** Marlo Vernon, Steven S. Coughlin, Martha Tingen, Samantha Jones, Vahe Heboyan

**Affiliations:** ^1^ Department of Medicine, Georgia Cancer Center, Cancer Prevention, Control, and Population Health, Medical College of Georgia Augusta University Augusta Georgia USA; ^2^ Department of Population Health Sciences, School of Public Health Augusta University Augusta Georgia USA; ^3^ Department of Family Medicine, Medical College of Georgia Augusta University Augusta Georgia USA

**Keywords:** African Americans, breast cancer, cancer prevention, cancer screening, cigarette smoking, colon cancer, health equity, obesity, prostate cancer, racial disparities, screening

## Abstract

**Background:**

The Cancer Health Awareness through screeNinG and Education (CHANGE) initiative delivers cancer awareness education with an emphasis on modifiable risk factors and navigation to screening for prostate, breast, and colorectal cancers to residents of public housing communities who experience significant negative social determinants of health.

**Methods:**

Residents of five communities participated. Community advisory board members were recruited and provided feedback to local environmental change projects, recruitment, and community engagement at each site. At each site, four education sessions were provided by trained facilitators on cancer risk factors and etiology, racial disparities, eligibility for cancer screening, and participation in clinical trials. Attendance, knowledge, attitudes and beliefs about cancer, and height, weight, and waist circumference were measured at baseline and 1‐week post‐CHANGE sessions.

**Results:**

90 residents (60% 65 and older years old, 33% male, 60% High School education, 93% AA) participated in the program. 95% completed post‐intervention evaluation. Participants were eligible for breast (*n* = 12), prostate (*n* = 15), and colorectal screening (*n* = 25) based on American Cancer Society guidelines, and 22 for tobacco cessation; 21 participants accepted navigation assistance for these services. At post‐test, participants significantly increased in knowledge and behaviors around obesity/overweight risk for cancer, nutrition, and physical activity. Colorectal, prostate, and breast cancer knowledge scores also increased, but were not significant.

**Conclusions:**

CHANGE participants demonstrated improved health knowledge and intentions to improve their modifiable health behaviors. Participants reported being motivated and confident in seeking preventive care and satisfaction with community engagement efforts. Replication of this project in similar communities may improve knowledge and health equity among underserved populations.

## 1 INTRODUCTION

Cancer is the second leading cause of death in the United States following heart disease.^1^ In the United States, at least 42% of newly diagnosed cancers are potentially avoidable, including 19% caused by smoking and 18% that are caused by a combination of excess body weight, physical inactivity, excess alcohol consumption, and poor nutrition.^1^ African Americans and other ethnic and racial populations in the United States suffer disproportionately from cancer.^1^ While overall incidence and mortality rates have decreased substantially for prostate, breast and colorectal cancers over the last few decades, African American men and women continue to suffer disproportionately. African Americans are less likely than whites to survive 5 years after being diagnosed with most forms of cancer, at any stage of diagnosis.^1^ African Americans also have the highest death rate and worse survival rate of any racial/ethnic group in the United States for most cancers. In addition, African American men and women are less likely to be screened, more likely to utilize lower‐resourced and non‐accredited facilities, and more likely to experience longer intervals between screening and follow‐up for abnormal findings.^2^, ^3^, ^4^

To reduce cancer disparities among African Americans, we developed and tested a multicomponent intervention. The Cancer Health Awareness through screening and Education (CHANGE) initiative was designed to improve cancer care and reduce racial disparities and inequities in a sustainable and collaborative community‐based manner by: (1) increasing evidence‐based cancer awareness though health literacy and education, with an emphasis on prevention of modifiable risk factors, screening, and early detection; and (2) providing access and navigation to high‐quality cancer screening and early detection services. Pre‐ and post‐intervention knowledge, attitudes and beliefs were measured, as well as changes in cancer health behaviors and rates of screening.

## 2 METHODS

The study population consisted of African American Augusta Housing Authority residents who were 21 to 80 years of age, English speaking, and able to answer questions on a computer or paper survey, either independently or with assistance. Up to 30 participants were recruited at each of five sites that delivered the intervention. The Theory of Planned Behavior was used a guiding framework for the project, with knowledge, beliefs (worry about cancer), and intentions (plans) assessed.

### 2.1 Recruitment

In collaboration with Community Advisory Boards (CABs) of representatives from each participating community, recruitment flyers were posted in community rooms and in community mailboxes. Staff attended community resident events including farmer's markets, back to school fairs, and holiday events to promote the study. Participants were also referred by the Director of the Augusta Housing Authority.

### 2.2 Community Advisory Board

A CAB was convened with 10 members representing the 5 respective Augusta Housing Authority sites. Members were elected representatives from each community's resident board who agreed to participate. Two CHANGE initiative personnel also served as members to help facilitate the twelve CAB monthly meetings. The CAB members received a small stipend for participation in promoting the program, completing community assessments, providing input on changes to create environments supportive of healthy lifestyle behaviors, and on strategies to overcome barriers to receiving cancer screening and follow‐up care.

### 2.3 CHANGE intervention

**FIGURE 1:**
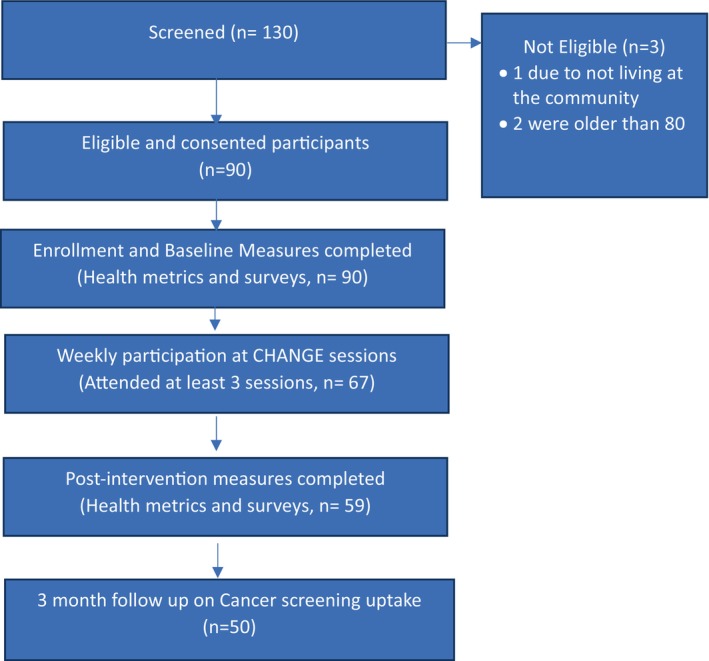
Flow diagram of study process.

The CHANGE intervention was composed of four education sessions completed in a group setting and facilitated by trained research staff (Figure 1). One session was presented weekly for four weeks. Each session lasted approximately 90 minutes. Facilitator and participant manuals were reviewed by the CAB and content experts; and were written at an 8th grade literacy level. Facilitators are trained research staff (n = 4, 100% female, 50% non‐White, all facilitators had at least a bachelors in a health‐related field) who assisted in the development of the program materials and received education on effective teaching methods. A facilitator's guide with scripts for each session at each site were also provided and utilized for fidelity to the program.

Sessions were held in a classroom or community room at each respective site. Two days prior to each session, intervention participants received either a reminder call or text reminder (based on personal preference). A virtual option was also made available for participants, involving self‐directed learning with the participant manual and online video tutorials.

Participants received a physical participant manual provided to them at the initial session. Each session was facilitated by trained staff, and began with a review followed by the presentation of a new topic lasting approximately 30 minutes. After each session, project team members called any absent participants to assess barriers to attendance, review any missing material, and remind them of the upcoming session.

Session 1 *Fight for your Life, what is cancer?* The CHANGE program was introduced, and information shared about what cancer is, common causes, incidence and prevalence was defined, and relevant local and state numbers were presented, risk factors for cancer, and their individual cancer risk were also presented and discussed. The American Cancer Society screening recommendations for various cancers, including the benefits of genetic testing was also presented. Tobacco cessation resources were shared, and current smokers were also navigated to Georgia Cancer Center's evidence‐based cessation services. The session ended with a guided family health history to evaluate familial genetic cancer risk.

Session 2 *Prostate Cancer, Breast Cancer, and Colorectal Cancer*. Participants learned about prostate, breast, and colorectal cancer, the benefits of early detection, signs and symptoms, and treatment options. Participants also learned about specific cancer incidence, prevalence, and disparities. Specific genetic, behavioral, and environmental risk factors and risk factor reduction for themselves and their families were also discussed.

Session 3 *Obesity and overweight as risk factors for cancer*. Obesity and overweight as risk factors for cancers and risk reducing behaviors for participants and their families were also discussed. The impact of nutrition and physical activity on reducing cancer risk and the impact of early childhood development of overweight will be presented, along with strategies and tools to develop healthy lifestyle behaviors.

Session 4. *Cancer Myths & Cancer Care Connections*. Participants will learn common myths about cancer. Supportive community resources for cancer patients and their families will be presented. All participants received a printed copy of the CHANGE resource guide for this module. The concept of navigation was introduced. Progress in cancer treatment with an emphasis on minority participation in clinical trials was also highlighted. This session ended with an interactive game that summarized key points of all sessions. Participants developed a personal action plan related to risk reduction and screening.

### 2.4 Assessment of Eligibility for Cancer Screenings

As part of the first session, participants completed a guided family health history to assess familial risk of cancer (immediate and extended family history of cancer, cancer type, and age at diagnosis if known) and completed simple screening questions to evaluate eligibility for prostate, breast, and CRC screenings. All eligibility guidelines utilized the screening criteria defined by the American Cancer Society when evaluating eligibility for screening.^5^ Prostate cancer screening eligibility was determined by: AA men 45 and older who have not discussed screening with a doctor, family history of prostate cancer before age 65, and with the following questions: “Have you ever had a Prostate Specific Antigen (PSA) blood test?” (yes/no), “If yes, when was your most recent one?” (less than or 1 year ago, more than 1 year ago). Breast cancer screening eligibility was determined by: women 45 or older who have not had a mammogram in the last year with the following questions: “Have you ever had a mammogram?” (yes/no), If yes, when was your most recent one? (Less than 1 year ago, between 1 and 5 years ago, or more than 5 years ago). Colorectal cancer screening was determined by the following criteria: 45 through 75, older than 75 with a recommendation of their physician, those who have not had a colonoscopy in the last 10 years or a FIT (fecal immunochemical test) in the last year, with the following questions: “Have you ever had a colonoscopy?” (yes/no); “If yes, when was your most recent one?” (less than 1 year ago, between 1 and 5 years ago, between 6 and 10 years ago, more than 10 years ago).

In addition, participants completed a self‐assessment of their personal cancer risk factors, adapted from the American Cancer Society's Lifestyle Behaviors and Risk Factors quiz (currently, this is available at cancer.gov as part of the Crucial Catch initiative). Participants who identified 3 or more risk factors (including family history, tobacco use, dietary habits, obesity, family history and sedentary lifestyle) were considered “high risk.”

If screenings were appropriate, participants were flagged for further follow‐up. Eligible participants were offered referrals to screening by project staff. Family history indicative of other cancers resulted in personalized recommendations. High risk participants were contacted by study staff, and referral options discussed and offered. A letter outlining their self‐reported risk factors was provided to take to their primary care physician. Three‐month follow‐ups were conducted for participants with high risk factors or who were due for a screening, to evaluate whether follow‐up care had been accessed.

Those who accepted referrals were provided eligibility about cancer screening materials provided by the Georgia Cancer Center, Cancer Information and Awareness resources. These resources utilized National Cancer Institute and American Cancer Society resources. These were provided along with a letter from the study to discuss with their primary care physician. If the participant did not have a primary care physician, they were referred with permission to the Georgia Cancer Center (GCC) patient navigators, who could also aid with scheduling and transportation. Participants were also provided information on accessing cancer screenings – this included availability of clinics in the Augusta area, information about when and where to go for breast, prostate, and colorectal screening, and information about what to expect at each screening (this was also discussed in session two).

### 2.5 Assessment of Eligibility for Tobacco Cessation

Participants self‐identified current smoking and/or other tobacco use. Current tobacco users were encouraged to quit. Those who agreed were referred to the GCC tobacco cessation services, a no cost evidenced‐based tobacco treatment program that includes four sessions (once a week) of counseling services and pharmacotherapy for 6 to 12 weeks depending on the level of nicotine addiction. Three months after the intervention, these individuals were contacted by telephone to determine if they had quit (or were in the process of quitting) and if there were any additional needs they may have had to be successful in quitting.

### 2.6 Follow‐up

Three months after the intervention, individuals flagged for cancer screening were contacted to determine if they had either obtained appropriate screening or discussed the screening with their healthcare provider. If a participant indicated a desire for cancer screenings but lacked the resources to pay for it, additional contact was facilitated with the with the GCC patient navigators to identify local resources to assist them, including the GCC and the Georgia Department of Public Health.

### 2.7 Data Collection

At baseline (one to two weeks prior to classes starting) and within one week of completing the educational sessions, participant data was collected at each respective site. This included sociodemographic data (baseline only) and relevant health information such as cancer history and cancer risk factors including history of tobacco use, as well as information about cancer screening history. This information allowed us to determine whether the participant was a potential candidate for prostate, breast, or colorectal cancer screening.

At baseline and immediate post‐test, we assessed participants' cancer knowledge, attitudes and beliefs concerning cancer, personal cancer‐related health behaviors such as smoking, healthy eating and physical activity behaviors, self‐efficacy, perceived cancer risk and cancer fears, and current awareness of cancer screening recommendations (adapted from the National Cancer Institute's *Health Information National Trends Survey* [*HINTS*]).^6^ Questions about breast cancer knowledge were adapted from the CDC's “Breast Cancer Quiz”,^7^ and the American Cancer Society Breast Cancer Quiz (https://www.cancer.org/cancer/types/breast‐cancer/breast‐cancer‐quiz.html),^8^ colorectal cancer knowledge questions were adapted from the CDC “Colorectal Cancer Quiz” (https://www.cdc.gov/cancer/colorectal/quiz/index.html),^9^ and prostate cancer knowledge survey items were used from the American Cancer Society, “Prostate Cancer Quiz”.^10^ Obesity and overweight knowledge questions were adapted from information in the National Cancer Institute's Obesity and Cancer page.^11^ Participant satisfaction with course materials was also assessed (post‐test only).

All participants had their height, weight, waist and hip circumference measured at baseline and post‐intervention by trained staff. All measures were repeated at least twice and retained within the standard of error. An average of the two metrics was retained for analysis. Body mass index (BMI) and waist/hip circumference were calculated as obesity screening measures. All surveys were created in Flexicapture to allow for scannable data entry of questionnaires and biometrics. Participants received a $25 gift card after completing baseline assessments and a $50 gift card when completing the post‐intervention assessments.

### 2.8 Statistical Analysis

The statistical analysis focused on evaluating the pre and post‐intervention changes in participant knowledge and behaviors related to cancer prevention, risk factors, and screenings. Data was evaluated pre/post, to evaluate a hypothesized increase in increase in knowledge and positive health behaviors after completing the program. Paired T‐tests, Chi‐Square, and ANOVA tests were used. All statistical tests were two‐tailed and used a significance level of 0.05.

The study was approved by the Augusta University Institutional Review Board and all participants completed informed consent prior to enrollment.

## 3 RESULTS

### 3.1 Demographics

Ninety participants completed informed consent and baseline measures (Table 1). A majority of the participants were African/Back American (88%). Six percent of the participants were younger than age 45 years, 31% between 45 and 64, and 57% were older than 65 years. Forty‐seven percent of participants had completed high school, 12% had only completed middle school and 41% reported some or more college education. Almost 80% of participants were overweight or obese, and 24% reported tobacco use. Eighty percent of participants attended at least 3 of the four sessions.

**TABLE 1:** Demographics (*n* = 90).

Sex	
Male	33 (33%)
Female	57 (64%)
Race	
African American/Black	84 (93%)
White	5 (6%)
American Indian/Alaska Native	1 (1%)
Age	
45 and younger	6 (7%)
46–64	30 (33%)
65 and older	54 (60%)
Education	
Less than high school	9 (10%)
High school/GED	54 (60%)
Greater than high school	25 (28%)
Prefer not to answer	2 (2%)
BMI	
Underweight	1 (1%)
Normal	14 (19%)
Overweight	16 (22%)
Obese	41 (57%)
Tobacco use	
Tobacco user	22 (24%)
Non‐user	68 (76%)
Site participation	
Site 1	20
Site 2	5
Site 3	18
Site 4	21
Site 5	26

At baseline, 58% (*n* = 41; 26 females and 15 males) of participants reported ever having a previous colon cancer screening, 77% of women had had at least one mammogram, and only 27% of males reported ever having a PSA screening. Among female participants, 59.5% reported plans to be checked for colon cancer at baseline and 73% reported plans at follow‐up (*χ* ^*2*^ = 2.490, *p <* 0.11); for males, 81.5% reported plans at baseline and 80% reported plans at follow‐up (*ns*).

### 3.2 Referrals for screening

After completing cancer screening eligibility questionnaires during the program sessions, 46 participants were identified as eligible for a breast, prostate, or colorectal cancer screening, genetic cancer risk due to family history, or for tobacco cessation (Table 2.). Of these, 21 participants chose to have a referral made on their behalf to their primary care physician or the Georgia Cancer Center Of those eligible for cancer screening, 42% participants requested a referral for breast cancer screening, 13% for prostate screening, and 40% for colorectal cancer screening – some participants received more than one referral. Additionally, participants eligible for a screening reported speaking to their primary care physician about breast (50%), prostate (33%), and colorectal cancer screenings (36%). All participants who reported tobacco use were referred for tobacco cessation services – one participant died before completing tobacco cessation.

**TABLE 2:** Eligibility and referral for cancer screening.

	Breast	Prostate	CRC	Tobacco
Eligible for screening	12	15	25	22
Referral requested	5	2	6	6
Reported discussing with PCP	6	5	9	3
Screening/cessation scheduled	2	2	4	7
Screening/cessation completed	1	2	3	5
PCP did not recommend screening	2	1	2	–

At the 3‐month follow‐up, 6 reported discussing a mammogram with their PCP, and of those, 3 had completed or had one scheduled. Two reported that their PCP did not recommend screening (reasons were not known). Five participants had discussed a prostate screening with their provider, four had either scheduled or completed one, only one did not have a screening recommended by their PCP. Nine participants reported discussing a colonoscopy or FIT test and of these, 7 had scheduled or completed one (5 males, 2 females); 2 were not recommended for screening by their PCP. One male participant reported a positive FIT result and was working with a patient navigator for follow‐up. Of the 22 participants who reported current smoking or tobacco use at baseline, only 17 reported still using tobacco products at post‐test.

### 3.3 Knowledge, Beliefs, and Intentions Surveys

A total score was created for each knowledge section of the survey, resulting in a percent correct. One third of participants demonstrated improved knowledge of breast cancer risk and screening information, although this was not significant; men showed greater improvement in knowledge than women (Table 3). Twenty‐two percent of female respondents felt that getting a mammogram would be easier for them after participation. Overall, knowledge about prostate cancer risk factors and screening types also improved, but not significantly; there was no difference between men and women (Table 4.). No significant improvement was observed for colorectal cancer knowledge among all participants; women demonstrated greater improvement than men, but the difference was not significant (Table 5). Paired t‐tests showed significant improvement for obesity/overweight knowledge questions between visit 1 and visit 2 (Mean improvement in score = 23.9%, *t* = −4.495, *p* < 0.001) (Table 6). Women demonstrated greater improvement in scores than men (28% vs. 17%, NS). Age and education categories were not significantly different between groups on knowledge questions.

**TABLE 3:** Breast cancer knowledge.

	Percent correct	
	Pre‐test (%)	Post‐test (%)
Most women who get breast cancer die from it	83	81
The best way to find breast cancer early is with a mammogram	89	96
Surgery is the only way to treat breast cancer	61	62
Men can get breast cancer	81	91
A mammogram is a vaccine	80	87
Family history can increase risk of breast cancer	74	77
Only women over the age of 65 get breast cancer	89	89
How often should women over 40 get mammograms	88	85
Total score	81	84

**TABLE 4:** Prostate cancer knowledge.

	Percent correct	
	Pre‐test (%)	Post‐test (%)
Men of any age can get prostate cancer	85	77
Prostate cancer often causes men to have trouble passing urine	83	95
Prostate cancer is very common in the US	94	93
Prostate cancer always needs to be treated right away	91	91
All men should be tested for prostate cancer	83	86
Nothing that can be done to lower your chances of getting prostate cancer	79	80
Most men diagnosed as having prostate cancer die of something else	51	40
Men are more likely to die of prostate cancer than because of heart disease	57	45
It is possible to have prostate cancer if a man does not have any symptoms	76	69
Prostate cancer is one of the least common cancers among men	51	53
If a man has an abnormal PSA test result, the doctor may recommend a prostate biopsy	83	87
The PSA test will find all prostate cancers	24	29
A prostate biopsy can tell you with more certainty whether you have prostate cancer	83	84
Loss of sexual function is a possible side effect of prostate cancer	83	78
Problems with urination are possible side effects of prostate cancer	75	76
The risk of developing prostate cancer increases with age	77	89
The risk of developing prostate cancer is higher in African American men than other racial or ethnic groups	83	100
Diet rich in fruits is likely to reduce risk for developing pros	63	67
Total Score	68	69

**TABLE 5:** Colorectal cancer knowledge.

	Percent correct	
	Pre‐test (%)	Post‐test (%)
Colorectal cancer is the second leading cancer killer in the U.S.	83	79
Getting screened for colorectal cancer can help you prevent the disease.	83	78
If you don't have any symptoms, it means you don't have colorectal cancer	78	73
The only screening test for colorectal cancer is colonoscopy	41	50
Medicare and most insurance plans cover colorectal cancer screening	87	83
Blood in or on your stool (bowel movement) is a symptom of colorectal cancer	63	65
Stomach pain, aches, or cramps that don't go away are a symptom of colorectal cancer	54	61
Losing weight and you don't know why, is a symptom of a colorectal cancer	52	61
Who gets colon cancer? (Men/Women/Both)	88	91
Screening is recommended to start at what age?	51	42
At what age can you stop getting screened for colorectal cancer?	69	58
Total Score	70	70

**TABLE 6:** Overweight and obesity knowledge.

	Percent correct	
	Pre‐test (%)	Post‐test (%)
Being overweight or obese puts one at higher risk of cancer	75	98
Minority persons have more cancer related to obesity.	67	81
Too much body fat can increase growth hormones, which can cause cancer	77	92
Adults are considered obese if they weight 30 or 40 pounds above their ideal weight	79	94
As far as you know, does being overweight or obese affect the risk of developing the following types of cancer?		
Breast cancer	32	72
Prostate cancer	39	65
Colon cancer	43	69
Brain cancer	23	37
Stomach cancer	38	62
Gallbladder cancer	33	54
Liver cancer	33	58
Kidney cancer	40	58
Multiple myeloma (Blood) cancer	27	46
Pancreatic cancer	31	63
Uterine or ovarian cancer	25	51
Total score	43	69

At baseline, 12% of females reported “often,” or “always” worry about getting breast cancer, and 14% of males reported worry about getting prostate cancer. 28% of males and 12% of females reported worry about getting colon cancer. At post‐test, 19% of females reported worry “often,” or “always,” about getting breast cancer, 14% of males worried about prostate cancer; 10% of males and 14% of females reported worry about getting colon cancer.

At baseline, 83% of females reported an intention to get a mammogram and 11% were undecided; at post‐test, 89% of women intended to get a mammogram and 7% were undecided. 81% of males and 59.5% of females indicated an intention to be checked for colon cancer. At post‐test, 73% of females and 81% of males reported intentions to access colon cancer screening. These differences were not significant.

### 3.4 Changes in Health Behaviors

At pre‐test, 10 respondents reported current smoking behaviors (whether it was cigarettes, e‐cigarettes, or other forms of tobacco). At post‐test, 3 of those participants reported no longer smoking. In total, six participants were referred to the Georgia Cancer Center Tobacco Cessation Clinic. The decrease in smoking behaviors was a significant difference (*χ* ^2^ = 34.163, *p* < 0.001).

Respondents reported a significant increase in daily consumption of more than three servings of fruit (14% to 18%, *χ* ^2^ = 16.078, *p* < 0.003) servings; those who reported more than three servings of vegetables (20%) per day remained the same between pre and post.

Significantly more respondents reported physical activity at least once a week at post‐test (58% vs 78%), (*χ* ^2^ = 5.979, *p* < 0.014). More respondents reported physical activity on three or more days per week at post‐test (52.1%) compared to pre‐test (44.7%) (*χ* ^2^ = 35.627, *p* < 0.017).

## 4 DISCUSSION

This four‐week education intervention, coupled with navigation to screening and referral for care significantly improved participants’ knowledge about cancer, attitudes and beliefs concerning cancer, personal cancer‐related health behaviors such as smoking, health eating and physical activity behaviors, self‐efficacy, perceived cancer risk and cancer fears, and current awareness of cancer screening recommendations. We found that women showed greater improvement in cancer knowledge compared to men regarding overweight and obesity risk with cancer, colorectal cancer, and men showed greater improvement in knowledge related to breast cancer information. This is important as African‐American men and women are a greater risk of having late diagnosed breast, prostate, and colorectal cancer and men typically do not adhere to screening guidelines when compared to women, as demonstrated in our qualitative results (Vernon, 2022). The findings that age and education were not associated with changes in knowledge may be due to the fact that our sample was more homogenous in other social determinants of health than larger samples outside of the public housing community setting.

Participants demonstrated significant improvements in modifiable risk factors and behaviors for cancer – tobacco use and obesity through changes in nutrition and physical activity. This is particularly significant as this program only offered education, but no direct intervention on these modifiable behaviors.

Study limitations included accessibility issues and self‐report metrics. At some sites, it was difficult to recruit sufficient participants who could participate at our meeting times, which were determined by building space and Augusta Housing Authority staff availability. For some participants, we were able to conduct follow‐up sessions individually with facilitators and we also provided at least two sessions of the program at each site. All participants received the written educational guide at the conclusion of the program for reference and referral. During the pandemic, we developed a virtual component of the education program, yet, most did not have access to devices or technical knowledge to watch videos online or answer survey questions in a digital platform. However, this pivot has allowed us to create an almost completely digital version of the program for future dissemination and expansion of the project.

Reports of uptake of cancer screening were self‐reported and not independently verified, as the study did not have access to participant medical records. Acceptability of the education sessions was not formally measured. However, we have informally collected evidence of acceptability through participant feedback and requests to return to the communities to repeat the program.

Our results demonstrate that education and navigation to care can improve both knowledge and behavior through culturally responsive methods and tools. Previous studies in public housing communities focused primarily on tobacco cessation and air quality improvement to impact asthma among adults and children.^12^, ^13^, ^14^, ^15^, ^16^ A previous community‐based participatory research project successfully sought to increase walking minutes among public housing residents.^17^ Similar to our study, participants increased in both nutrition and physical activity self‐report and days of activity. Another study sought to increase CRC screening, physical activity, and multivitamin use in public housing residents, and reported that residents were interested in more education on cancer screening.^18^ Residents of these communities are often from underserved racial, minority, and financial groups.

Interventions of this type and intensity are particularly beneficial to underserved and marginalized communities who experience significant disparities due to socioeconomic and access issues. By increasing knowledge, improving attitudes and beliefs, and providing tools to encourage conversation with providers, more eligible adults are equipped with the tools to be active participants in their preventive health care. These results support the implication that knowledge builds trust – exemplified by the increase in participants reporting conversations with their providers about accessing cancer screenings. Similar populations are not often reached by traditional or mainstream educational outreach evidenced by a lack of access to the internet and computer devices. Simple replicable, readable, and personal educational outreach has the additional benefit of improving trust among populations who often do not have confidence that they will receive quality care. Training community health workers to deliver the curriculum will also increase sustainability in the program. Future expansion of this project into other residential communities including public housing, mixed housing, and retiree communities may also improve underserved community knowledge about cancer, cancer risks, and their access to care.

## PRECIS

The Cancer Health Awareness through screeNinG and Education (CHANGE) initiative is designed to deliver cancer awareness education, with an emphasis on modifiable risk factors, and navigation to screening for prostate, breast, and colorectal cancers to residents of public housing communities who experience significant negative social determinants of health. CHANGE participants demonstrated improved health knowledge and intentions to improve their modifiable health behaviors.

## AUTHOR CONTRIBUTIONS

**Marlo Vernon:** Conceptualization (equal); data curation (equal); formal analysis (equal); funding acquisition (equal); investigation (equal); methodology (equal); project administration (equal); supervision (lead); writing – original draft (lead); writing – review and editing (lead). **Steven S. Coughlin:** Funding acquisition (supporting); writing – original draft (equal); writing – review and editing (equal). **Martha Tingen:** Conceptualization (supporting); funding acquisition (supporting); methodology (supporting); project administration (supporting); writing – review and editing (supporting). **Samantha Jones:** Methodology (supporting). **Vahe Heboyan:** Data curation (supporting); formal analysis (supporting); funding acquisition (supporting); methodology (supporting); writing – review and editing (supporting).

## FUNDING INFORMATION

This project was funded by Pfizer and the American Cancer Society through the *Addressing Racial Disparities in Cancer Care* call.

## CONFLICT OF INTEREST STATEMENT

The authors have no conflicts of interest to report.

## Supporting information


Appendix S1.

## Data Availability

The data that support the findings of this study are available from the corresponding author upon reasonable request.
