# Supplementary material for: Cancer health awareness through screening and education: A community approach to healthy equity
Source: Cancer Med. 2024 Jun 28;13(13):e7357. doi: 10.1002/cam4.7357 (PMC11211999; doi:10.1002/cam4.7357)
Supplement: Supplementary file 1 — Appendix S1. [file CAM4-13-e7357-s001.zip › plain language summary Augusta.docx]

The Cancer Health Awareness through screeNinG and Education (CHANGE) initiative provides cancer awareness education, help with getting cancer screenings, and information about risk factors for cancer that can be changed – obesity and overweight and smoking. We provided this program to public housing residents over a 2 year period. Participants improved on their knowledge about cancer and reported increased healthy behaviors – including more fruits and vegetables in their diets and more physical activity during the week. They also received referrals and help getting cancer screenings they were eligible for. Providing similar outreach programs in the community is an important step to prevention and early diagnosis of cancer among vulnerable populations.
