# Supplementary material for: Cancer health awareness through screening and education: A community approach to healthy equity
Source: Cancer Med. 2024 Jun 28;13(13):e7357. doi: 10.1002/cam4.7357 (PMC11211999; doi:10.1002/cam4.7357)
Supplement: Supplementary file 1 — Appendix S1. [file CAM4-13-e7357-s001.zip › short summary Augusta.docx]

The Cancer Health Awareness through screeNinG and Education (CHANGE) initiative is designed to deliver cancer awareness education, with an emphasis on modifiable risk factors, and navigation to screening on prostate, breast, and colorectal cancers to residents of public housing communities. Participants demonstrated improvements in knowledge about cancer risks, cancer risk reducing health behaviors, and screening rates.
